# Milk-derived peptone reduces EHEC virulence by inhibiting the type III secretion system

**DOI:** 10.1080/21505594.2026.2721084

**Published:** 2026-09-05

**Authors:** Dor Braverman, Albert Batushansky, Neta Sal-Man

**Affiliations:** a The Shraga Segal Department of Microbiology, Immunology, and Genetics, Faculty of Health Sciences, Ben-Gurion University of the Negev, Beer-Sheva, Israel; b The Ilse Katz Institute for Nanoscale Science & Technology, Ben-Gurion University of the Negev, Beer-Sheva, Israel

**Keywords:** Bacterial virulence, casein, diet, *Escherichia coli*, peptone, type 3 secretion system

## Abstract

The link between diet and overall human health is well established, with certain diets known to promote better health and be associated with a reduced risk of developing chronic diseases. Previous research has also demonstrated the direct effects of diet on microbiome composition, diversity, and fitness, which can, in turn, alter colonization resistance and the immune response against enteric pathogens. However, much less is known regarding the direct effect of diet on the virulence of pathogenic bacteria. To examine the effect of diet on bacterial virulence while maintaining constant macronutrient composition, we used peptones from different sources (plant- and animal-based) to simulate various dietary protein sources. The peptones were examined for their effect on the virulence of enterohemorrhagic *Escherichia coli* (EHEC). We found that bacteria grown with peptone derived from casein – the main protein component of milk – exhibit a significant reduction in their type III secretion system (T3SS) activity. This effect manifested as a significant reduction in bacterial adherence to host cells and limited translocation activity of the T3SS effector Tir. We additionally found evidence suggesting that milk-derived peptone causes a shift in bacterial behavior from hyper-virulent, adherent bacteria to a more motile state. Finally, we observed that the inhibitory effects of milk-derived peptone extend beyond EHEC to other T3SS-possessing pathogens. While the specific inhibitory components in casein remain to be identified, our findings provide a foundation for developing casein-based, non-antibiotic therapies against bacterial diarrheagenic pathogens.

## Introduction

Diet plays a crucial role in human health by providing the essential nutrients, vitamins, and minerals necessary for optimal physiological function. Dietary choices have a direct impact on intestinal function and immune response, linking nutrition to chronic diseases such as cardiovascular diseases, diabetes, and certain cancers [1–3]. A poor diet can lead to nutrient deficiencies, inflammation, and metabolic dysfunction, whereas a balanced, nutritious diet supports optimal body function and helps prevent disease.

Beyond its direct effects on the host, diet also influences health through its impact on the gut microbiome [4–8]. For example, diets rich in fiber and plant-based foods promote the growth of beneficial bacteria such as *Bifidobacteria* and *Lactobacilli* that enhance gut-barrier integrity and immune modulation [9]. In contrast, high-fat and low-fiber Western diets tend to reduce microbial diversity and favor the expansion of potentially harmful taxa [10]. Given that the microbiome provides colonization resistance against invading pathogens by competing for space and nutrients, diet-induced changes to its composition can profoundly affect pathogen colonization and disease progression [11]. Additionally, certain microbiome species can produce protective metabolites from the breakdown of dietary components. These include short-chain fatty acids (SCFAs), such as acetate and butyrate, as well as primary and secondary bile acids like taurocholate and deoxycholate. These protective metabolites have been shown to suppress pathogens’ virulence by reducing the expression of genes related to bacterial adhesion, motility, invasion, and toxin production [12–15]. The concentrations of these protective metabolites can be altered directly by dietary intake, which provides precursors for their production, or indirectly by altering the microbiome composition that has the genetic capacity for secondary bile acid and SCFA production.

While the influence of diet on the immune response and microbiome-mediated colonization resistance is well-studied, the direct effects of specific dietary components on bacterial virulence remain poorly understood. Nevertheless, emerging evidence suggests that certain dietary factors can modulate bacterial virulence and, in turn, affect disease outcomes. For example, *Helicobacter pylori*, a pathogen linked to severe gastric diseases, has been shown to upregulate virulence genes in response to high salt concentrations [16]. This observation is further supported by a study of *H. pylori*-infected gerbils, where bacteria from animals fed a high-salt diet exhibited increased expression of virulence genes compared to those on a regular diet [17]. In contrast, high salt concentrations showed the opposite effect on *Vibrio cholerae*, the causative agent of cholera, where elevated salt levels in the growth medium suppressed the expression of virulence genes [18]. Other dietary compounds, such as oligosaccharides, have been shown to reduce bacterial colonization and infection [11]. For instance, galacto-oligosaccharides (GOS), commercially produced from lactose using glycoside hydrolases, have been shown to reduce the adherence of enterohemorrhagic *E. coli* (EHEC) to tissue culture cells [19]. Similarly, administration of chitosan oligosaccharide has been shown to reduce *V. cholerae* intestinal colonization and disease severity in mice [20]. In addition, a recent study demonstrated that while the microbiome can limit the ability of an enteric pathogen to establish infection by actively depleting amino acids, consumption of a protein-rich diet neutralizes this protective effect [21].

Despite these important findings, our understanding of how particular dietary ingredients directly influence bacterial virulence remains limited. Therefore, in this study, we investigated whether dietary proteins from different sources modulate the virulence of EHEC, a foodborne pathogen that can cause severe gastrointestinal illness, including bloody diarrhea and hemolytic uremic syndrome (HUS) [22]. EHEC belongs to the family of attaching and effacing (A/E) pathogens, which includes enteropathogenic *E. coli* (EPEC) and the mouse pathogen *Citrobacter rodentium*. This group of pathogens is characterized by its tight adherence to the intestinal epithelial cells and the formation of a unique morphology, resembling a pedestal, under the attached bacteria. The pedestals are formed through cytoskeleton rearrangements within the infected host cell, resulting in the effacement of microvilli and the formation of dense actin filaments beneath the attached bacteria. To mediate bacterial attachment and pedestal formation, A/E pathogens utilize their type III secretion system (T3SS) [23,24]. This system resembles a nano-syringe and enables the translocation of various bacterial proteins, termed effectors, into the cytoplasm of the host cells. The bacterial effectors subvert various cellular processes to support bacterial survival and replication [25]. In A/E pathogens, the T3SS is encoded on a ~ 35-kbp chromosomal pathogenicity island, called the locus of enterocyte effacement (LEE) [26,27]. Another enteric pathogen that utilizes T3SS to promote infection is *Salmonella enterica* serovar Typhimurium (referred to as *S*. Typhimurium), a major human gastrointestinal pathogen that occupies a similar intestinal niche but causes disease through a distinct invasive infection strategy [28]. Unlike EHEC, *Salmonella* does not produce A/E lesions or induce pedestal formation on intestinal epithelial cells. Instead, *S*. Typhimurium encodes two distinct T3SSs located on Salmonella Pathogenicity Islands 1 and 2 (SPI-1 and SPI-2), with SPI-1 mediating epithelial cell invasion and SPI-2 enabling survival and replication within intracellular vacuoles [28–30].

Here, we characterized EHEC’s virulence response to various protein sources (termed peptones) that are derived from plant or animal origins, focusing on T3SS activity, bacterial adherence, and host-cell infection.

## Materials and methods

### Bacterial strains

The strains used in this study are listed in Table 1; EHEC O157:H7 strain EDL 933, EHEC O157:H7 strain 86–24 [nalidixic acid-resistant], EPEC O127:H6 strain E2348/69 [streptomycin-resistant], *Citrobacter Rodentium* DBS100 [ampicillin-resistant], and *Salmonella enterica* serovar Typhimurium SL1344 [streptomycin-resistant]. The bacteria were grown in Luria-Bertani (LB) broth (Sigma) supplemented with the appropriate antibiotics [streptomycin (50 μg/mL), ampicillin (100 µg/mL), or nalidixic acid (50 μg/mL)], at 37°C, with shaking.

**Table 1.** Bacterial strains used in this study.

Strain	Description	Reference
EHEC EDL 933	EHEC O157:H7 EDL 933	[31]
EHEC 86–24	EHEC O157:H7 86–24, Nalidixic acid resistant	[32]
EPEC	EPEC O127:H6 E2348/69, streptomycin resistant	[33]
*Citrobacter rodentium*	*C. rodentium* DBS100, ampicillin resistant	[34]
*Salmonella* Typhimurium	*Salmonella* Typhimurium SL1344, streptomycin resistant	[35]

### Media preparation

Peptone water (1% w/v) was prepared in phosphate buffer (Na_2_HPO_4_ / KH_2_PO_4_, pH 7.5) containing sodium chloride (85.6 mM). The following peptone sources were used: Bovine milk-derived peptone (casein-derived – also called tryptone; Formedium Limited, Santa Cruz Biotechnology, MilliporeSigma, or Gibco ThermoFisher Scientific), meat-derived peptone (Formedium), pea-derived peptone (Sigma-Aldrich), soy-derived peptone (Formedium), or casamino acid solution (BD Biosciences).

### Bacterial growth

Bacteria were grown overnight in LB broth at 37°C, with shaking. The overnight culture was then inoculated into a pre-warmed 1:1 (v/v) mixture of Dulbecco’s modified Eagle’s medium (DMEM – without phenol red) and peptone water. Bacterial growth was assessed at 37°C by measuring the OD_600_, with cultures shaken at 30-minute intervals immediately before each measurement. At least three independent experiments were conducted, and the average values are presented.

### In vitro type III secretion (T3S) assay

T3S assays were performed as previously described [36,37]. Briefly, bacteria were grown overnight in LB broth supplemented with the appropriate antibiotics at 37°C, shaking. The overnight cultures were then washed with PBS, diluted 1:40 into a pre-warmed 1:1 (v/v) mixture of DMEM and peptone water, and grown statically for 6 h (EPEC & *Cirtobacter rodentium)* or 8 h (EHEC) in a tissue culture incubator (with 5% CO_2_). To separate the bacterial pellets from the supernatants, the cultures were centrifuged at 20,000 × *g* for 5 min. The bacterial pellets were dissolved in SDS-PAGE sample buffer, and the supernatants were collected and filtered through a 0.22-μm filter (Millipore). Cultures’ OD_600_ values were used to normalize the supernatants, which were then precipitated with 10% (v/v) trichloroacetic acid (TCA) overnight at 4°C in order to concentrate the secreted proteins found in the culture medium. The TCA-treated supernatants were then centrifuged at 18,000 × *g* for 30 min at 4°C, the precipitates were resuspended in SDS-PAGE sample buffer, and saturated Tris was used to neutralize the residual trichloroacetic acid. Secreted (supernatant) and bacterial (pellet) proteins were analyzed by SDS-PAGE, followed by Coomassie Blue staining (InstanBlue, Abcam Inc.) or western blot analysis.

To examine T3SS activity of bacteria grown under anaerobic conditions, overnight cultures were washed and diluted into a pre-warmed 1:1 (v/v) mixture of DMEM and peptone water and grown statically for 8 h in an anaerobic chamber (Don Whitley A35 anaerobic work station; 5% H_2_, 10% CO_2_, and 85% N_2_). The samples were processed and analyzed as described above for the aerobic conditions.

The T3SS activity of *Salmonella* pathogenicity island 1 (SPI-1) was determined as described previously [38]. Briefly, overnight *Salmonella* cultures were washed and diluted 1:40 into peptone water supplemented with 4 mM glucose and grown at 37°C for 4 h, with shaking. The supernatant and pellet samples were prepared as described above, with precipitated supernatant samples being subjected to an additional cold acetone wash following the TCA treatment and prior to dissolvement in SDS-PAGE sample buffer.

### Western blotting

Samples were subjected to SDS-PAGE and then transferred to nitrocellulose membrane (pore size, 0.45 µm; Cytiva) or PVDF membrane (pore size, 0.45 µm; Cytiva) using a similar protocol as described in [36]. The membranes were blocked for 1 h with 5% (w/v) skim milk in PBST (0.1% (v/v) Tween in phosphate-buffered saline) for the duration of 1 h and then incubated with the primary antibody (diluted in 5% skim milk/PBST) for 1 h at room temperature or overnight at 4°C. The membranes were thereafter washed, and then incubated with the secondary antibody (diluted in 5% skim milk/PBST) at room temperature for 1 h. Chemiluminescence was detected with WESTAR-ECL reagents (Cyanegen). The following primary antibody dilutions were used: rabbit anti-JNK (Abcam Inc.), diluted 1:1000; mouse anti-DnaK (Abcam, Inc.), diluted 1:5000; and mouse anti-Actin (MPBio), diluted 1:10,000. Antibodies directed against *E. coli* T3SS components were a generous gift from Prof. Rebekeh DeVinney (University of Calgary, Canada) and included rat anti-Intimin, rat anti-Tir, and rat anti-EspD. The antibody against the *Salmonella* SigD effector was a generous gift from Prof. B. Brett Finlay (University of British Columbia, Canada). The horseradish peroxidase conjugated (HRP)-goat anti-mouse (Abcam Inc.), HRP-conjugated goat anti-rabbit (Abcam Inc.), and HRP-conjugated goat anti-rat (Abcam Inc.), were used as secondary antibodies at a 1:10,000 dilution. Representative western blots of at least three independent experiments are presented in the Results section.

### Translocation assay

Translocation assays were performed as previously described [39] with some modifications. Briefly, bacteria were grown for 3 h under T3SS-inducing conditions (DMEM only or in a 1:1 (v/v) mixture of DMEM and peptone water, at 37°C, static, in a tissue culture incubator). HeLa cells (8 × 10^5^) were infected with the primed bacteria at a multiplicity of infection (MOI) of ~300. Infections were carried out for 3 h in DMEM only or in a 1:1 (v/v) mixture of DMEM and the various peptone waters. Cells were thereafter washed and incubated with fresh media containing gentamicin (100 µg/mL) for 30 min to remove non-attached bacteria from the cultures. Cells were then washed with cold PBS and lysed with RIPA buffer. Samples were then centrifuged at 18,000 × *g* for 5 minutes to remove non-lysed cells, and supernatants were collected. The samples were resuspended in SDS-PAGE sample buffer, boiled, and subjected to western blot analysis with anti-JNK, anti-Tir, and anti-Actin (loading control) antibodies. Uninfected cells were used as a negative control, and a sample of bacterial pellet was included to show Tir in its unmodified form.

### Bacterial adhesion assay

Overnight bacterial cultures were washed with PBS and diluted 1:40 into pre-warmed DMEM or a 1:1 (v/v) mixture of DMEM and peptone water. Cultures were then incubated statically for 3 h in a tissue culture incubator (with 5% CO_2_) to induce T3SS activation. The primed bacteria were used to infect HeLa cells (1 × 10^5^) at an MOI of ~100 and incubated for 2 h in the corresponding media. Cells were then thoroughly washed with PBS to remove unattached bacteria and lysed by incubation with 0.1% (v/v) Triton for 15 min at room temperature. Cell lysates were collected, serially diluted in sterile PBS, plated on LB plates, and incubated at 37°C overnight. The colony forming units (CFUs), representing the intimately-associated bacteria, were counted. Each condition was tested in triplicate, with uninfected samples serving as negative controls.

### RNA extraction and qPCR analysis

RNA extraction and qPCR assays were performed as previously described in [38]. Briefly, wildtype EHEC overnight cultures were washed with PBS and diluted 1:40 into pre-warmed DMEM or a 1:1 (v/v) mixture of DMEM and milk-derived peptone (tryptone) water, and grown statically in a tissue culture incubator (with 5% CO_2_) for 6 h. Bacteria were then collected, resuspended in lysis buffer (50 mM glucose, 25 mM Tris pH 7.5, 10 mM EDTA), and incubated with lysozyme (0.5 mg/mL, Sigma) for 10 minutes at 37°C. RNA was extracted using TRIzol reagent (Invitrogen), treated with DNase I (NEB), and purified using a second round of TRIzol extraction. For cDNA synthesis, 1 μg of RNA was reverse transcribed by ProtoScript II First Strand cDNA Synthesis Kit (NEB) using a random primer mix. The resulting cDNA was examined for genomic DNA contamination before further processing. The qPCR reactions were performed using SYBR Green I mix (Roche) and analyzed in the QuantStudio cycler (Applied Biotechnologies, Thermo). The reaction conditions for amplification were: 1 cycle at 95°C for 10 min, 40 cycles of 95°C for 15 s, cooling to 58°C for 15 s, followed by 72°C for 15 s while monitoring fluorescence. The sequences of the primers used for the qPCR experiments are presented in Table 2. Melting curve analysis was used to validate the specificity of each primer pair. Data were analyzed by QuantStudio software to extract the critical threshold (*C*_*T*_) values. The transcription levels of the target genes under different growth conditions were normalized to the *16S* rRNA gene and compared using the relative quantification method. Real-time data are presented as the fold change in transcription levels.

**Table 2.** Primers used in this study.

Primer name	Sequence	Reference
16S_qPCR_F	CTTACGACCAGGGCTACACAC	This study
16S_qPCR_R	CCGTGGCATTCTGATCCACG	This study
ler_qPCR_F	GACTGCGAGAGCAGGAAGTT	This study
ler_qPCR_R	CCAGGTCTGCCCTTCTTCATTG	This study
tir_qPCR_F	CGCCTGTAAGGAATTCTATGGC	This study
tir_qPCR_R	GCCTGTTAAGAGTATCGAGCGG	This study
espB_qPCR_F	GCTTCGGAGAGTACGACCG	This study
espB_qPCR_R	CGGCCTGCTGAATCTGATAGC	This study
espD_qPCR_F	CAGTTGTCCCGGCAGAACG	This study
espD_qPCR_R	ATGGGGGAAAATGCCCCTC	This study
eae_qPCR_F	GCACTTAATGCCAGTGCGG	This study
eae_qPCR_R	ACTGTCGCACTAACAGTCGC	This study
ehxA_qPCR_F	GGAAACAGTGACGCACATACAGG	This study
ehxA_qPCR_R	AGCCAGCATAACAGCCGATG	This study
fliC_qPCR_F	ACTAACAACGCTGGTAGCGC	This study
fliC_qPCR_R	CCGCTTTGGTTTCGCTCAATAC	This study
flhD_qPCR_F	TGCATACCTCCGAGTTGCTG	This study
flhD_qPCR_R	GCGTGTTGAGAGCATGATGC	This study
stx1_qPCR_F	AACCGCCCTTCCTCTGGATC	This study
stx1_qPCR_R	GCTGAATGTCATTCGCTCTGC	This study
stx2_qPCR_F	CTGTTAATGCAATGGCGGCG	This study
stx2_qPCR_R	ACTTCAGCAAATCCGGAGCC	This study

### Motility assay

Overnight bacterial cultures were collected, washed, and inoculated onto semi-solid agarose motility plates (0.3% (w/v) agarose), containing either DMEM only or a 1:1 (v/v) mixture of DMEM and milk-derived peptone or casamino acid solution, final concentration 0.5% (w/v). The plates were incubated in a tissue culture incubator (with 5% CO_2_) for 8 h. The non-motile bacterium *C. rodentium* was used as a negative control. Motility was calculated as the ratio between the measured radii of the migration ring and the inoculation ring. Each condition was performed in triplicate. The bars present the average of at least three independent experiments.

### LC-MS analysis of the media

The Ultra Performance Liquid Chromatography (UPLC) system (Waters Corp.) coupled with high-resolution mass-spectrometer (MS) Exploris 240 (Thermo Fisher Scientific Inc.) was used for the analysis of the media sources: casamino acid solution, milk- and meat-derived peptones. The samples were first dissolved in LC-MS grade water (0.1 g/mL), shaken for 10 min at 20°C, centrifuged (5 min, 20°C, 18,000 × *g*) and filtered through 0.2 µm filter. 100 µL of the upper phase were transferred to the new tube with 2 µL of 40 µg/mL internal standard (13C15N-phenylalanine, Cambridge Isotopes) and 398 µL of prechilled LC-MS grade methanol. The samples were shaken for 2 minutes at 4°C and centrifuged for 10 min at 4°C, 18,000 × *g*. 10 µL from the top layer were collected to the new tube and mixed with 990 µL of LC-MS grade water. Lastly, 40 µL of the samples were mixed with 60 µL of LC-MS grade acetonitrile, transferred into the glass vials and subjected to the LC-MS analysis along with the standard amino acid mix (Cat#79248, Sigma). Each sample type was prepared in three replicates independently. A separation of the compounds was achieved on BEH amide column (Waters Corp.) using a gradient of 10 mM ammonium format, pH 3.5 in 90% water (mobile phase A) and 90% acetonitrile (mobile phase B) for 25 min. The injection volume was 1 µL, the column temperature was kept at 30°C, and the flow rate was 0.25 mL/min. High-resolution mass spectra were acquired using electron spray ionization (ESI) in positive full scan mode (60–950 *m/z*) with a resolution of 24,000 full width at half-maximum (FWHM). The MS parameters were: ion spray voltage −3,500, sheath gas −50, aux gas −1, ion transfer tube temperature −325°C, and vaporizer temperature −350°C. Data were acquired under the control of Xcalibur software version 4.5.455 (Thermo Fisher Scientific Inc.) (https://www.thermofisher.com).

The data analysis was performed using Xcalibur Quan Browser. First, the processing method for amino acids was created using a standard mixture and internal standard to identify their detected m/z and retention times. Next, all data were processed using this method to identify the compounds and integrate their peak areas. Last, a relative abundance of each target was calculated by ratio between its peak area and internal standards peak area, providing a relative abundance of each target in the samples.

### Pedestal formation assay

Overnight bacterial cultures of WT EPEC were collected, washed, and diluted 1:20 in PBS. HeLa cells (3.5 × 10^4^ seeded on glass coverslips) were infected with 3 µL of the diluted culture either in DMEM only or a 1:1 (v/v) mixture of DMEM and milk-derived peptone. Infections occurred in a tissue culture incubator (with 5% CO_2_) for 3 h. After infection, cells were thoroughly washed with PBS to remove unattached bacteria, fixed in 4% paraformaldehyde for 15 min, and permeabilized with 0.2% (v/v) Triton X-100 for 5 minutes at room temperature. Cells were then incubated in blocking solution (5% (w/v) bovine serum albumin (BSA) and 0.1% (v/v) Triton X-100 in PBS) overnight at 4°C. Next, samples were incubated with the rabbit anti *E. coli* primary antibody (Abcam), diluted 1:2000 in blocking solution, at room temperature for 1 h, washed (0.1% (v/v) Triton X-100 in PBS), and incubated with Alexa fluor 488-conjugated goat anti-rabbit IgG secondary antibody (Abcam), diluted 1:1000 in blocking solution, for 1 h at room temperature. Following additional washes, cells were stained with TRITC-labeled phalloidin (0.2 µg/mL, Sigma) for 15 min, washed, and mounted using Fluoro-Gel II mounting medium with DAPI (Bar-Naor). Samples were imaged using an Axio Observer inverted fluorescence microscope equipped with a 63× oil immersion objective and controlled by ZEN Pro software (ZEISS, Oberkochen, Germany).

## Results

### EHEC T3SS activity is inhibited by milk-derived peptone

**Figure 1.**
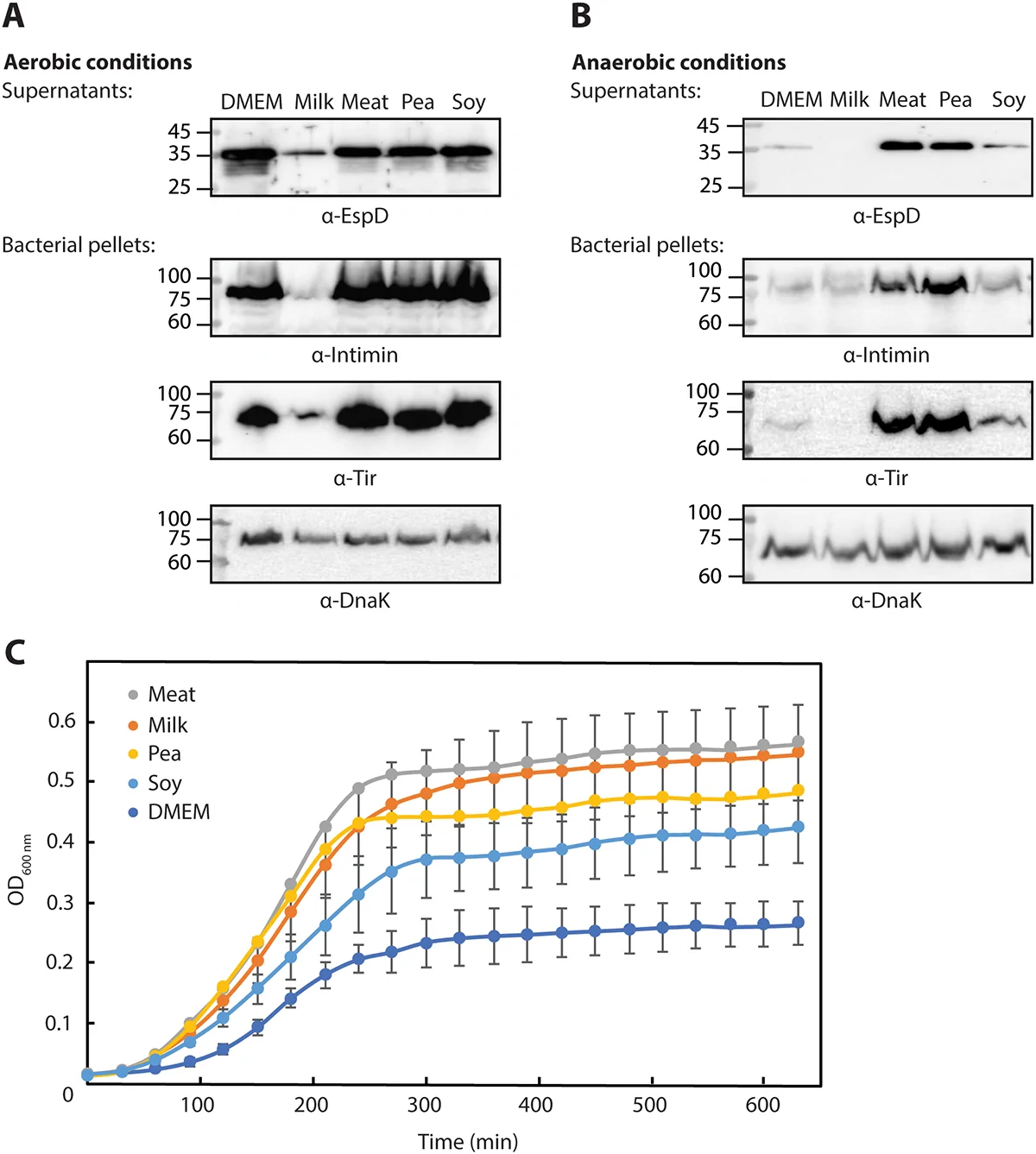
Milk-derived peptone suppresses the T3SS activity of EHEC. Protein secretion profiles of EHEC EDL933 grown under T3SS-inducing conditions in the presence of peptones from different origins (milk, meat, pea, or soy) at aerobic (A) and anaerobic (B) Conditions. The secreted fractions were normalized according to OD_600_, concentrated from the supernatants of bacterial cultures, and analyzed by western blotting using an anti-EspD antibody. Bacterial pellets were analyzed for the expression of Intimin and Tir. DnaK was used to verify an equal load of bacterial material. Blots are representative of at least three independent experiments. (C) Growth curves of EHEC grown in DMEM alone or a 1:1 (v/v) mixture of DMEM and different peptone waters (originated from milk, meat, pea, or soy). Data are the means of at least three independent experiments; bars represent standard deviation.

To determine the effect of dietary components on bacterial virulence, we cultured EHEC (O157:H7 strain EDL 933) in the presence of peptone, a product generated from the enzymatic hydrolysis of proteins. Peptone can be produced from a variety of sources, including milk, meat, pea, soy, or other protein-rich substrates. These peptones contain peptides, amino acids, and inorganic salts as well as other compounds, such as lipids, vitamins, and sugars [40]. As such, different peptone sources can represent various protein-based diets in a relatively defined and controlled manner. For plant-based protein sources, we selected pea and soy peptones, and for animal-based protein sources, we used meat and milk (also known as tryptone) peptones. To evaluate the effect of peptone source on EHEC virulence, we assessed T3SS activity, a key virulence mechanism of this pathogen. EHEC was grown under T3SS-inducing conditions in the presence of various peptones (milk, meat, pea, and soy). The cultures were then centrifuged to separate the bacterial pellets (indicating protein expression) and their supernatants (indicating protein secretion). The samples were analyzed by SDS-PAGE and western blotting using antibodies against T3SS components. EHEC growing in the presence of meat-, pea-, and soy-derived peptones secreted EspD at comparable levels as when grown in DMEM only, the standard medium for assessing EHEC T3SS activity. In contrast, growth in the presence of milk-derived peptone led to a marked reduction in EspD secretion (Figure 1(A)). Moreover, the expression of the Tir effector and the outer membrane adhesin Intimin was significantly reduced in the presence of milk-derived peptone, whereas no such reduction was observed with other peptones (Figure 1(A)). Since oxygen availability varies dramatically along the gastrointestinal tract and plays a key role in regulating EHEC virulence [41,42], we assessed the impact different peptones on T3SS activity during anaerobic growth. As expected, EHEC grown in DMEM alone under anaerobic conditions displayed reduced T3SS activity compared to aerobic growth conditions (Figure 1(B)), consistent with previous reports showing that low-oxygen conditions suppress expression and maturation of the EHEC T3SS [41–43]. Notably, supplementation with meat-, pea-, or soy-derived peptones increased T3SS activity relative to anaerobic growth in DMEM alone (Figure 1(B)), indicating that nutrient-rich peptones can partially counteract the suppressive effect of anaerobiosis on T3SS activation. In contrast, milk-derived peptone maintained its inhibitory effect on T3SS activity, as reflected by minimal EspD secretion and reduced Tir and Intimin expression (Figure 1(B)).

To determine whether this effect is specific or observed in other EHEC isolates, we performed a similar T3SS assay with an additional clinical EHEC strain (O157:H7 86–24). We found that milk-derived peptone has a similar suppression effect on the T3SS activity of EHEC 86–24 (Figure S1). To ensure that T3SS suppression was not specific to milk-derived peptone from a single supplier, we tested multiple milk-derived peptones from different vendors. We observed that the T3SS activity was consistently inhibited in the presence of milk-derived peptones compared with DMEM alone (Figure S2), indicating that this is a general effect of milk-derived peptone. While the samples in the T3SS assay are normalized according to OD_600_ values, we wanted to confirm that the effect of milk-derived peptone was unrelated to impaired bacterial growth. To that end, we grew EHEC in 1:1 (v/v) mixtures of DMEM (without phenol red) and peptone waters and recorded bacterial growth over time (Figure 1(C)). As expected, enhanced bacterial growth was observed in all mixtures of DMEM and peptones compared to DMEM alone, a medium generally considered nutrient-poor (Figure 1(C)). However, no statistically significant differences in bacterial growth were observed between the different peptone mixtures.

### Milk-derived peptone attenuates EHEC infection in vitro

**Figure 2.**
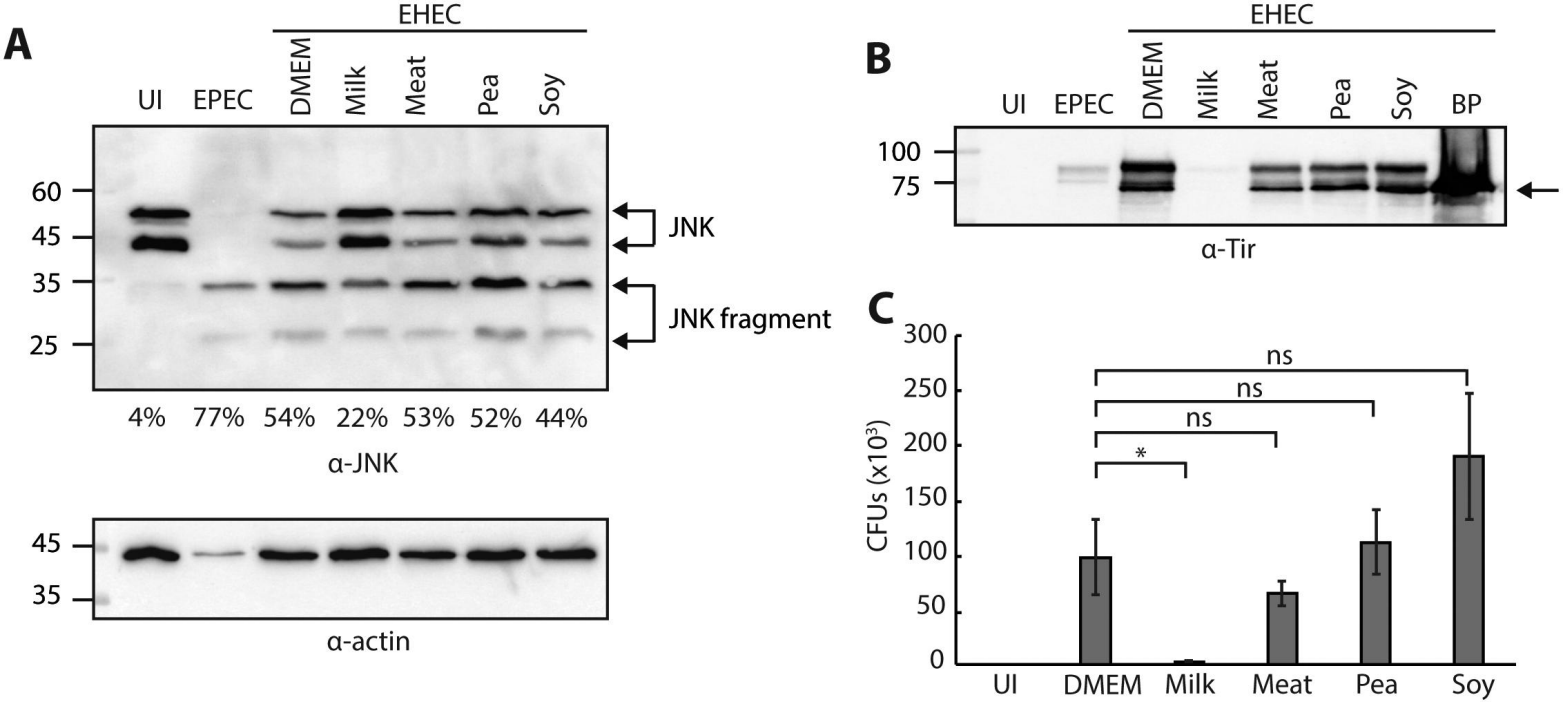
Milk-derived peptone hinders EHEC ability to infect cells *in vitro*. HeLa cells were infected with primed EHEC (pre-grown under T3SS-inducing conditions) in the presence of peptones from different origins (milk, meat, pea, or soy). At the end of the infection period (3 h), cells were washed and lysed, and the cellular lysates were analyzed for JNK degradation (A) as well as Tir translocation and modification (B). (A) JNK isoforms and degradation fragments are indicated at the right of the gel. The degree of JNK degradation (shown as % at the bottom of the JNK blot) was determined by calculating the ratio between the intensity (quantified by densitometer) of the JNK fragments and the total cellular JNK intensity. Cells infected by EPEC and uninfected cells serve as positive and negative controls, respectively. The membrane was also probed with anti-actin antibody to confirm equal sample loading. (B) To determine the level of Tir translocation from bacteria into host cells, cellular lysates were subjected to SDS-PAGE and analyzed with anti-Tir antibody. The higher molecular weight band corresponds to modified Tir that arises following protein translocation, whereas the lower band represents the unmodified Tir (labeled with an arrow). A sample of bacterial pellet (BP) was used as a control to demonstrate the unmodified bacterial Tir. Blots are representative of at least three independent experiments. (C) To evaluate bacterial adherence, HeLa cells were infected with EHEC, washed to remove non-adherent bacteria, lysed, and plated for CFU quantification. A representative experiment is presented; similar trends were obtained across three independent experiments. Bars represent standard deviation; *, *p* < 0.05, unpaired Student’s t-test. ns, not significant.

To further investigate the effects of the different peptones on EHEC virulence, we employed a modified version of the well-established *in vitro* infection model developed by Baruch et al [39]. We grew the bacteria in DMEM alone or in 1:1 (v/v) mixtures of DMEM and peptone water, both before and during the infection of HeLa cells. Infection efficiency was assessed by monitoring the cleavage of c-Jun N-terminal kinase (JNK), a cellular protein specifically cleaved by the translocated T3SS effector NleD [39]. Since this assay was originally developed for enteropathogenic *E. coli* (EPEC), another A/E pathogen, we included wildtype EPEC as a positive control. EPEC grown in DMEM alone induced extensive JNK degradation and cell detachment (as evident by the reduced actin signal) relative to the uninfected samples, whereas EHEC grown under identical conditions (marked DMEM in Figure 2(A)) showed a milder effect, with approximately half of the total cellular JNK content being cleaved (Figure 2(A)). These results established the JNK cleavage assay as a viable method to assess EHEC infection *in vitro*, though the weaker phenotype relative to EPEC confirms previous indications that EHEC does not infect cell lines as effectively as EPEC [44,45]. Nonetheless, comparison of EHEC grown in different peptone mixtures revealed that growth in meat-, pea-, and soy-derived peptones resulted in JNK cleavage levels similar to those observed in DMEM, whereas growth in milk-derived peptone led to attenuated cleavage (Figure 2(A)). Quantification of cleaved JNK as a percentage of total JNK confirmed this observation (percentages shown below the JNK blot). These findings complement the T3SS results, suggesting that the reduction in T3SS activity observed with milk-derived peptone impaired NleD translocation and consequently reduced JNK degradation.

Since NleD translocation by EHEC was found to be less informative than in EPEC, we also examined effector translocation into host cells. As Tir is the most efficiently translocated effector in A/E pathogens [46], we anticipated that its translocation would provide the greatest sensitivity. Upon its translocation into the host cell, it undergoes phosphorylation by the host kinases [23,47]. We analyzed lysates of infected cells using an anti-Tir antibody, which detected both unphosphorylated (~75 kDa, indicated by an arrow) and phosphorylated (~90 kDa) Tir. Both Tir forms were detected in lysates of cells infected with EPEC and with EHEC grown in DMEM, whereas no signal was observed in the uninfected cells (Figure 2(B)). Infection with EHEC grown in meat-, pea- and soy-derived peptones resulted in Tir levels comparable to those in DMEM, while growth in milk-derived peptone led to a pronounced reduction in Tir translocation (Figure 2(B), Table S1).

Tir translocation into host cells promotes intimate bacterial attachment to cells through its tight interaction with Intimin, a T3SS protein expressed on the bacterial surface [24]. This intimate attachment is a crucial step for successful gut colonization and the formation of the hallmark A/E lesions. We therefore examined the effects of various peptones on the ability of EHEC to adhere to host cells *in vitro*. To test this, we grew the bacteria in 1:1 (v/v) mixtures of DMEM and peptone water, both prior to and during infection of HeLa cells. Following infection, the cells were thoroughly washed and lysed. The resulting lysates contained cell-associated bacteria, a population expected to be associated primarily through Tir-Intimin dependent intimate adhesion, although additional Tir-independent adhesion mechanisms may also contribute. These lysates were plated for CFUs counting. Lysates collected from cells infected with EHEC grown in the presence of milk-derived peptone yielded significantly fewer CFUs than those infected with EHEC grown in DMEM only (Figure 2(C)). These results suggested that growth in the presence of milk-derived peptone reduced bacterial association with host cells, consistent with the observed decrease in Tir translocation. The CFU counts for cells infected with bacteria grown in the presence of meat-, pea-, and soy-derived peptones fluctuated but did not significantly differ from the CFU counts of bacteria grown in DMEM (Figure 2(C)).

### EHEC’s gene expression is altered by milk-derived peptone

**Figure 3.**
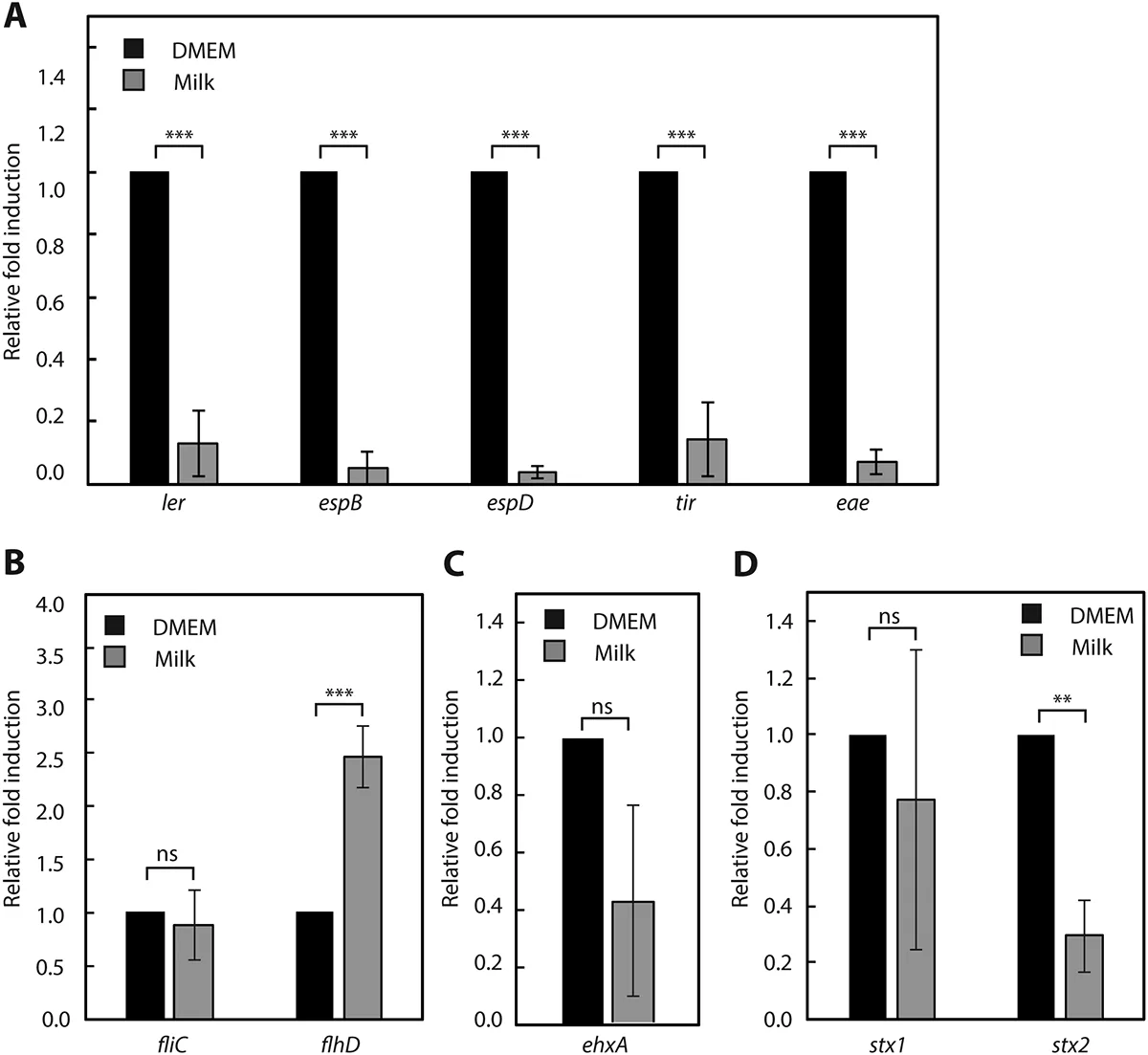
Milk-derived peptone alters EHEC gene regulation. EHEC strain EDL933 was grown under T3SS-inducing conditions in DMEM or in a 1:1 (v/v) mixture of DMEM and milk-derived peptone. Transcription levels of mRNA encoding (A) T3SS-related genes *(ler, espB, espD, tir*, and *eae*), (B) Motility-related genes (*fliC* and *flhD*), (C) The hemolysin gene, *ehxA*, and (D) stx coding genes (*stx1, stx2*), were measured by qRT-PCR. mRNA levels of EHEC grown in the presence of milk-derived peptone (gray bars) are presented relative to those of EHEC grown in DMEM only (black bars). Data are the means of at least three independent experiments; bars represent standard deviation; **, *p* < 0.01, ***, *p* < 0.005, unpaired Student’s t-test. ns, not significant.

Following the identification of a clear and consistent phenotype affecting T3SS activity and EHEC virulence only in the presence of milk-derived peptone, subsequent experiments were focused on this peptone. To better understand the mechanism by which milk-derived peptone suppresses EHEC T3SS activity, and to examine whether it is mediated via transcription regulation, we grew EHEC under T3SS-inducing conditions in DMEM alone or in a 1:1 (v/v) mixture of DMEM and milk-derived peptone. We extracted RNA from bacteria grown under the two growth conditions and examined the fold-change in mRNA levels of several virulence-related genes. For the T3SS, we examined the transcription of five representative genes: *ler*, the master regulator of the LEE operon; *espB* and *espD*, the two translocators that form the T3SS pore in the host membrane; *tir*, which encodes the first translocated effector; and *eae*, which encodes Intimin, the outer membrane adhesin that serves as the cognate receptor for Tir. We detected a significant reduction in the transcription levels of all five genes in bacteria grown in the presence of milk-derived peptone (Figure 3(A)). In addition, we examined the effect of milk-derived peptone on the transcription of motility-related genes; *fliC*, encoding the distal flagellar component, and *flhD*, the flagellar master regulator [48]. While *fliC* transcription showed no significant change, *flhD* transcription was significantly enhanced in the presence of milk-derived peptone (Figure 3(B)). When examining the effect on the transcription of *ehxA*, the gene encoding hemolysin [49], we observed a trend toward reduced transcription that was not statistically significant (Figure 3(C)). In addition, we evaluated the effect of milk-derived peptone on the transcription of the major EHEC virulence factor, Shiga toxin (Stx). Stx is a phage-encoded toxin that binds Gb3-expressing cells and inhibits protein synthesis by removing a conserved adenine residue from the 28S rRNA, contributing to cell cytotoxicity and the development of HUS [50,51]. While we observed no significant change in *stx1* transcription, *stx2* transcription was reduced approximately 3.4-fold in the presence of milk-derived peptone (Figure 3(D)). Taken together, these findings indicate that milk-derived peptone does not uniformly affect the gene regulation of EHEC virulence mechanisms.

### EHEC exhibits a hyper-motile phenotype in the presence of a milk-derived peptone and casamino acid solution

**Figure 4.**
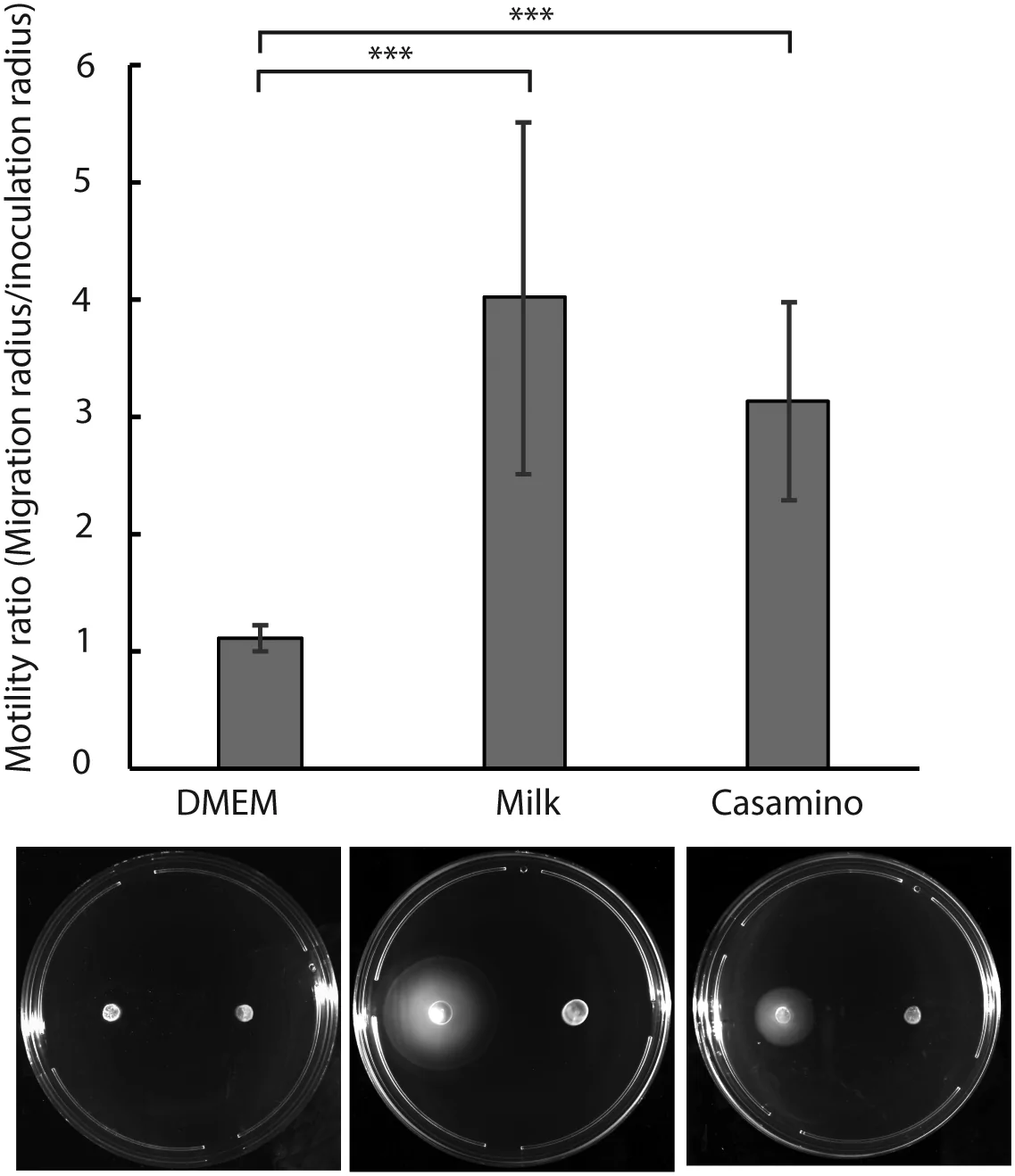
Milk-derived peptone and casamino acid solution enhance EHEC motility under T3SS-inducing conditions. EHEC was inoculated onto semi-solid [0.3% (w/v)] agarose plates containing DMEM or 1:1 (v/v) mixtures of DMEM and either milk-derived peptone or casamino acid solution, and incubated for 8 h at 37°C in a tissue-culture incubator. Motility is presented as the ratio between the migration and inoculation radii. Data are the means of at least three independent experiments; bars represent standard deviation; ***, *p* < 0.005, unpaired Student’s t-test. Representative images of each condition are shown beneath the corresponding bar. The inoculum to the right in each plate is the non-motile bacterium *C. rodentium*, which serves as a negative control.

Given our qPCR results and the reported mutual exclusivity between T3S and motility systems [48,52,53], we investigated whether milk-derived peptone induces a hyper-motility phenotype in EHEC. Additionally, to better understand which bioactive compounds in the milk-derived peptone contribute to this phenotype, we also tested a casamino acid solution. Similar to milk-derived peptone, casamino acids are derived from casein hydrolysis; however, milk-derived peptone contains mostly peptides, whereas acid hydrolysis of casein produces casamino acids that consist primarily of free amino acids and are depleted of certain micronutrients [54,55]. We cultured EHEC under T3SS-inducing conditions on semi-solid agarose media comprised of DMEM alone or 1:1 (v/v) mixtures of DMEM and either milk-derived peptone or casamino acid solution. These plates allow EHEC migration, which can be assessed by measuring the radius of bacterial spread around the inoculation point. We used the non-motile bacterium *C. rodentium* as a control to distinguish between passive diffusion and active motility. We observed that EHEC grown in the presence of either milk-derived peptone or casamino acid solution exhibited significantly enhanced motility compared to EHEC grown in DMEM alone (Figure 4). Casamino acids appeared to induce motility to a lesser extent than milk-derived peptone, though this difference was not statistically significant.

### Lysine and ornithine alone do not mediate the T3SS-suppressive effect of milk-derived peptone

**Figure 5.**
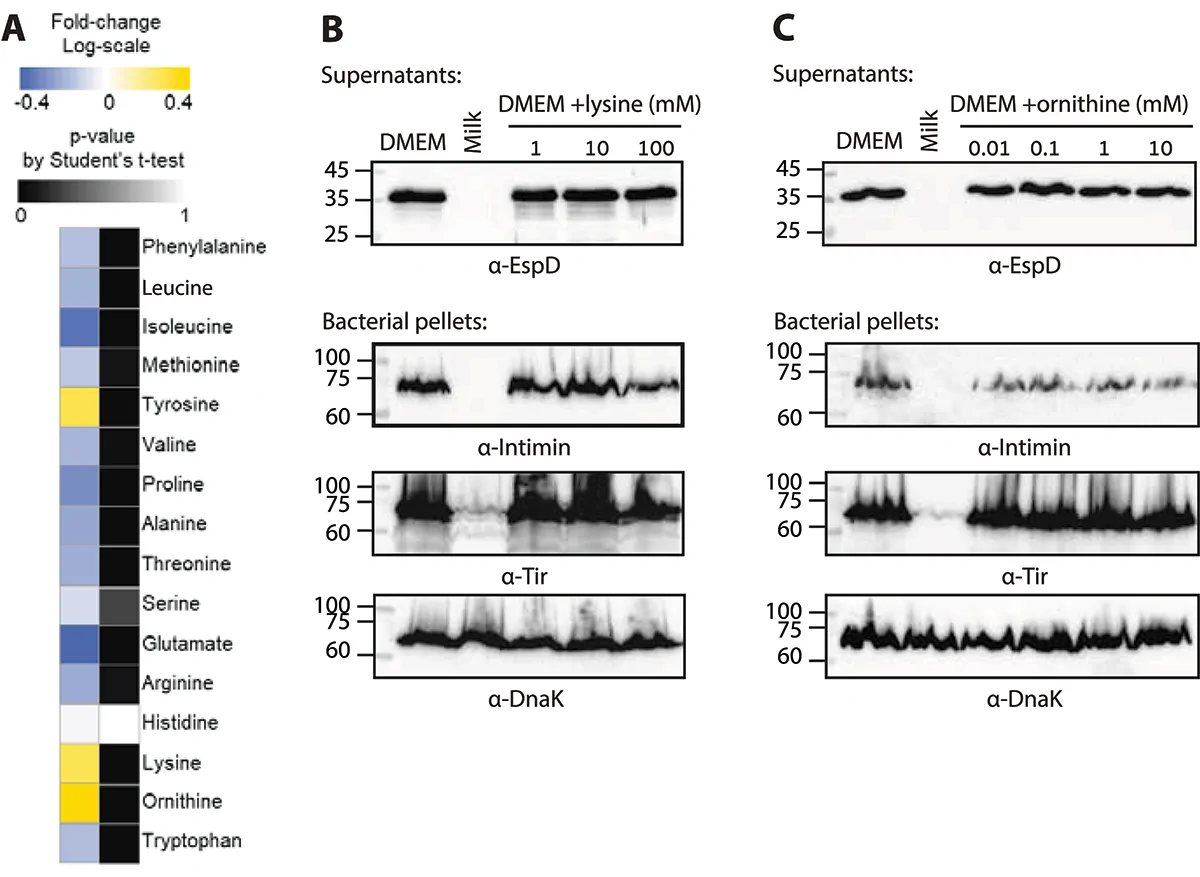
Single amino acids enriched in milk-derived peptone do not recapitulate the suppression of T3SS activity. (A) Heatmap showing the relative abundance of amino acids between milk- and meat-derived peptones. The left column displays the fold-change between milk and meat peptones, the right column shows the corresponding *p*-values (Student’s t-test, *n* = 3). (B-C) Protein secretion profiles of EHEC EDL933 grown under T3SS-inducing conditions with increasing concentrations of lysine (B) or ornithine (C). The secreted fractions were normalized according to OD_600_, concentrated from the supernatants of bacterial cultures, and analyzed by western blotting using an anti-EspD antibody. Bacterial pellets were analyzed for Intimin and Tir expression. DnaK was used to verify equal loading of bacterial material. Milk-derived peptone (milk) was included to demonstrate maximal suppression. Blots are representative of at least three independent experiments.

In light of the similar phenotypes observed when EHEC was grown in the presence of milk-derived peptone or casamino acids solution, we hypothesized that this effect might be mediated by one or more specific amino acids. To investigate this, we performed targeted LC-MS analysis of milk-derived peptone, casamino acids solution, and the non-T3SS-suppressive meat-derived peptone for comparison. The analysis detected 15 proteinogenic amino acids at markedly different abundances across the tested media, with most amino acids present at significantly higher levels in the casamino acids solution than in either peptone source, as expected (Table S2). A pairwise comparison between milk- and meat-derived peptones shows that, although most amino acids were more abundant in meat-derived peptone, lysine and tyrosine were approximately twofold higher, and the non-protein amino acid ornithine was approximately threefold higher, in milk-derived peptone (Figure 5(A)). Based on these differences, we examined the effects of lysine and ornithine on EHEC T3SS activity. Tyrosine was excluded due to its limited solubility under the tested growth conditions. EHEC cultures were grown in DMEM supplemented with increasing concentrations of lysine or ornithine, and secretion profiles were analyzed as described above. EspD secretion remained comparable between cultures grown in DMEM alone and those supplemented with either lysine (Figure 5(B)) or ornithine (Figure 5(C)), in contrast to the marked decrease observed in cultures grown in the presence of milk-derived peptone. Similar patterns were observed for Tir and Intimin expression (Figure 5(B,C)). Taken together, these results indicate that lysine or ornithine alone cannot recapitulate the T3SS-suppressive effect of milk-derived peptone.

### Milk-derived peptone and casamino acid solution suppress the T3SS activity of other pathogens

**Figure 6.**
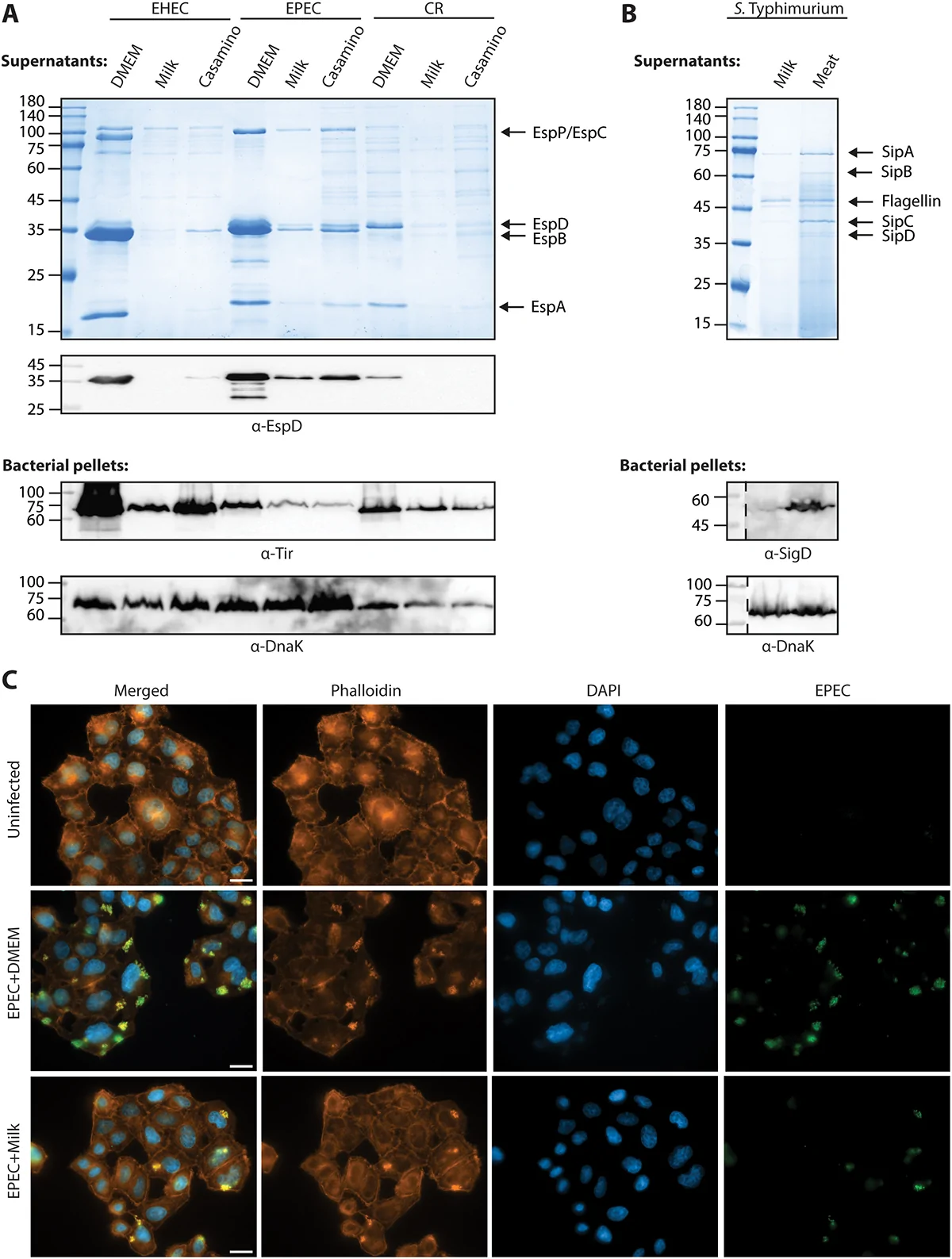
Milk-derived peptone and casamino acid solution suppress the T3SS activity of several T3SS-possessing pathogens. (A) Protein secretion profiles of A/E pathogens: EHEC, EPEC, and *C. rodentium* grown under T3SS-inducing conditions in the presence of milk-derived peptone or casamino acid solution. The secreted fractions were normalized according to OD_600_, concentrated from the supernatants of bacterial cultures, and analyzed by Coomassie stain (secreted proteins are indicated by arrows on the right side of the gel) and western blotting with an anti-EspD antibody. Bacterial pellets were analyzed for Tir expression. DnaK was used to verify equal loading of bacterial material. (B) Protein secretion profile of *S. enterica* serovar typhimurium grown under SPI-1-inducing conditions in milk- or meat-derived peptones. The samples were treated as described in panel A and analyzed by Coomassie stain (secreted proteins are indicated by arrows on the right side of the gel). Bacterial pellets were analyzed for expression of the effector SigD. Blots are representative of at least three independent experiments. (C) Fluorescence microscopy images showing actin pedestals formation on HeLa cells infected with WT EPEC in either DMEM alone or the presence of milk-derived peptone. Actin was stained with phalloidin (red), cell nuclei were stained with DAPI (blue) and bacteria were stained with Alexa 488 (green). Scale bar: 20 µM. Images are representative of at least three independent experiments.

To investigate whether the T3SS inhibitory effect of milk-derived peptone is specific to EHEC or extends to additional A/E pathogens, we examined two additional species: EPEC, a human pathogen, and *C. rodentium*, a murine pathogen. We cultured each under T3SS-inducing conditions in either DMEM alone or in a 1:1 (v/v) mixture of DMEM and milk-derived peptone for 6 h. Bacterial pellets (expression) and supernatants (secretion) were collected and subjected to SDS-PAGE followed by either Coomassie staining or western blot analysis using antibodies against T3SS components. Coomassie staining and western blot analysis revealed a significantly reduced secretion of T3SS translocators (EspA, EspB, and EspD) and reduced expression of the Tir effector in EPEC and *C. rodentium* grown with milk-derived peptone vs DMEM alone (Figure 6(A)). In addition, we tested whether casamino acid solution could recapitulate the T3SS suppression observed with milk-derived peptone by growing the pathogens in a 1:1 (v/v) mixture of DMEM and casamino acid solution. Notably, we observed a comparable reduction in T3SS activity in EHEC, EPEC, and *C. rodentium* cultured in the presence of casamino acid solution, indicating this response is mediated by low-molecular-weight compounds.

To extend our examination to non-A/E pathogens, we studied the effect of milk-derived peptone on *S*. Typhimurium. The SPI-1 T3SS of *S*. Typhimurium is functionally equivalent to the T3SS of EHEC. Since induction of the SPI-1 T3SS requires different conditions than those used for the previously tested pathogens, *S*. Typhimurium was cultured exclusively in milk-derived peptone water and compared with meat-derived peptone water, which did not suppress T3SS in EHEC. Replacement of milk-derived peptone with casamino acid solution did not support proper bacterial growth (data not shown) and was therefore not tested. We observed reduced amounts of secreted translocators in the supernatant of *S*. Typhimurium grown in milk-derived peptone water compared to meat-derived peptone (Figure 6(B)). In addition, the expression of the SigD effector was significantly reduced in bacterial pellets of bacteria grown with milk-derived peptone compared to meat-derived peptone. Finally, we examined the effect of milk-derived peptone on the ability of EPEC to form actin pedestals during infection, a hallmark feature of A/E pathogens that depends on a functional T3SS [56,57]. We infected HeLa cells with EPEC in either DMEM alone or in a 1:1 (v/v) mixture of DMEM and milk-derived peptone. After infection, we fixed and stained the cells, and assessed pedestal formation using an inverted fluorescence microscope. We observed robust pedestal formation for cells infected with EPEC in DMEM alone (Figure 6(C)). However, when cells were infected with EPEC in the presence of milk-derived peptone, we observed markedly reduced pedestal formation, with fewer adhered bacteria and microcolonies. These findings suggests that milk-derived peptone impairs EPEC’s pedestal-forming and adherence capabilities. Taken together, these results indicate that milk-derived peptone affects multiple T3SS-possessing pathogens, pointing to the involvement of a conserved mechanism.

## Discussion

The effects of diet on gut health have been widely investigated. This includes the effects of diet on the gut microbiome, which is highly susceptible to dietary changes [4]. However, the impact of diet on bacterial pathogens has been studied primarily in the context of the gut microbiome, rather than through the direct dietary influence on the pathogens. While a few studies have demonstrated direct effects of specific diet-derived metabolites on bacterial pathogens [58–60], this area remains largely unexplored, particularly in a broader nutritional context.

In this study, we characterized the effects of peptones derived from various protein sources on a key virulence factor of EHEC, the T3SS. While bacterial growth was comparable across plant- and animal-based peptones, milk-derived peptone significantly reduced T3SS activity relative to the other peptones and DMEM, an optimized T3SS-inducing medium (Figure 1(A)). This was further verified for EHEC grown under anaerobic conditions (Figure 1(B)) and for an additional EHEC clinical isolate (Figure S1). The effect was consistent for various milk-derived peptones from multiple vendors (Figure S2), thus confirming that milk-derived peptone can suppress T3SS activity. Using cell culture infection models, we found that T3SS suppression by milk-derived peptone translated into reduced infectivity (Figure 2), mediated through decreased transcription of T3SS-related genes (Figure 3).

The reduction in transcription of T3SS-related genes in the presence of milk-derived peptone was accompanied by additional gene expression changes, including a non-statistically significant reduction in transcription of the hemolysin-encoding gene ehxA. This observation is consistent with previous reports indicating that ehxA is only partially regulated by ler [49,61], whose transcription was significantly downregulated under these conditions. Although speculative, the absence of statistically significant downregulation of the plasmid-encoded ehxA may indicate the involvement of additional regulatory mechanisms, including plasmid-specific regulatory pathways.

The increased transcription of *flhD*, the master regulator of the flagella, was reflected phenotypically by enhanced motility of bacteria grown in the presence of milk-derived peptone compared to DMEM alone (Figure 4). These opposing effects of milk-derived peptone on the T3SS activity and the flagellar system are consistent with the well-established inverse regulatory relationship between LEE-encoded virulence functions and motility in EHEC [48]. Previous studies have shown that the GrlR-GrlA regulatory system coordinately controls LEE and flagellar gene expression; GrlA represses transcription of the flagellar master regulator operon *flhDC* while activating the LEE [52]. Likewise, the small RNA Esr41 inversely regulates LEE and flagellar genes [53]. Our observations that milk-derived peptone decreases T3SS activity but enhances motility and *flhD* transcription align well with these established regulatory relationships. This pattern suggests that milk-derived peptone affects upstream regulatory pathways, shifting bacterial behavior from a hyper-virulent, adherent state to a more motile phenotype. These results are consistent with previous studies demonstrating the anti-adhesive effect of milk-derived components on EHEC [62]. Additionally, the suppression of T3SS activity by milk-derived peptone in multiple enteric pathogens, including *S*. Typhimurium (Figure 6(B)), suggests that conserved metabolic and environmental regulatory pathways may be involved. In both A/E pathogens and *S*. Typhimurium, virulence-associated T3SS expression is tightly coordinated by global regulators such as H-NS, CsrA, and CRP-cAMP, as well as broader metabolic sensing networks that couple nutrient availability and environmental cues to virulence gene regulation [63–67].

Of note, while *stx1* transcription was not significantly affected, *stx2* transcription was reduced approximately 3.4-fold in the presence of milk-derived peptone. Although both toxins are prophage-encoded and responsive to the bacterial SOS response, *stx2* expression depends primarily on transcription from the phage late promoter following prophage induction, whereas *stx1* can also be transcribed from a ferric uptake regulator (Fur)-dependent promoter in response to iron availability [51,68,69]. Previous studies have shown that the Fur-dependent promoter is the dominant regulatory element controlling *stx1* expression, while transcription from the phage late promoter is not required for high-level Stx1 production [70]. Accordingly, the selective reduction in *stx2* transcription observed in our study (Figure 3(D)) is consistent with the distinct regulatory mechanisms governing *stx1* and *stx2* expression. These findings suggest that, under the conditions tested, milk-derived peptone does not promote regulatory conditions associated with increased *stx2* expression and may instead suppress its transcription. Together with the reduced transcription of T3SS-related genes, these results further support the hypothesis that milk-derived peptone affects a global regulator involved in the control of multiple virulence-associated systems. Importantly, this supports the potential value of milk-derived peptone as an anti-virulence treatment strategy. Nevertheless, additional studies under optimized toxin-inducing conditions will be required to fully assess its impact on Stx production.

The antibacterial activity of milk-derived components, particularly the protein fraction, against bacterial pathogens is well established. Most of these studies have focused on the antimicrobial activities of various casein-derived peptides, both from human and bovine sources, on pathogens such as *Staphylococcus aureus*, *Yersinia enterocolitica*, and diarrheagenic *E. coli* [71–74]. In contrast, we report here the anti-virulent activity of milk-derived peptone, rather than its general antimicrobial effects. Evidence for the anti-virulence activity of milk-derived peptides is limited, with most studies reporting the anti-adhesive effect of casein-derived peptides [75–77] or their inhibitory effect on biofilm formation, as exemplified in *Listeria monocytogene*s [78]. Our results reveal a novel anti-virulence mechanism in which milk-derived peptone and casamino acids solution can suppress the T3SS, a major virulence factor, without exerting bactericidal effects. Our results coincide with a recent study that demonstrated that mice fed a casein-based diet had significantly lower colonization levels of *V. cholerae* compared to mice fed soy-based or regular chow diets [54]. This effect was reported to be partially mediated by the downregulation of virulence-related genes encoding the type VI secretion system. These observations align with our findings that specific protein sources differentially affect bacterial virulence.

Interestingly, while most previous studies have focused on casein-derived peptides, as the active components of milk, our findings suggest that smaller compounds may mediate this effect, since the suppression of the T3SS activity persists even when bacteria are grown in casamino acid solution (Figure 6(A)). To explore the possibility that a specific milk-derived bioactive compound mediates this effect, we tested whether indole, a tryptophan derivative previously reported to inhibit T3SS activity in diarrheagenic *E. coli* [79,80], could be responsible. However, no significant differences in indole concentrations were detected between the different media (data not shown). To determine whether T3SS suppression could be attributed to specific amino acids present in the different peptones, we analyzed the amino acid profiles by targeted LC-MS analysis. Although lysine and ornithine were enriched in milk-derived peptone relative to meat-derived peptone, supplementation with either amino acid alone did not significantly alter EHEC T3SS activity, suggesting that the suppressive phenotype is likely mediated by other metabolites or by a synergistic effect of multiple components rather than by a single compound.

In summary, we report the inhibitory effect of milk-derived peptone on the T3SS activity of EHEC and additional enteric pathogens. Further work is needed to pinpoint the specific bioactive compounds responsible, but these findings highlight the potential of diet-based, non-antibiotic treatment strategies to combat diarrheal bacterial infections.

## Supplementary Material

Table S2.csv

Supplementary information.docx

Table S1.xlsx

## Data Availability

The authors confirm that the data supporting the findings of this study are available within the article, its supplementary materials. Raw data supporting all figures are openly available in Figshare https://doi.org/10.6084/m9.figshare.30995179 [81].

## Abbreviations

A/E: Attaching and effacing
EHEC: Enterohemorrhagic *Escherichia coli*
LEE: Locus of enterocyte effacement
T3SS: Type III secretion system
