# Supplementary material for: Milk-derived peptone reduces EHEC virulence by inhibiting the type III secretion system
Source: Virulence. 2026 Sep 5;17(1):2721084. doi: 10.1080/21505594.2026.2721084 (PMC13556935; doi:10.1080/21505594.2026.2721084)
Supplement: Supplementary file 1 [file KVIR_A_2721084_SM0835.docx]

Figure S1: **Milk-derived peptone suppresses the T3SS activity of an additional clinical EHEC strain**. Protein secretion profiles of EHEC 86-24 grown under T3SS-inducing conditions in the presence of peptones from different origins (milk, meat, pea, and soy) at aerobic (A) and anaerobic conditions (B). The secreted fractions were normalized according to OD_600_, concentrated from the supernatants of bacterial cultures, and analyzed by western blotting with an anti-EspD antibody. Bacterial pellets were analyzed for Intimin and Tir expression. DnaK was used to verify equal bacterial load.

Figure S2: **EHEC T3SS activity is suppressed by milk-derived peptone obtained from different vendors**. Protein secretion profiles of EHEC EDL933 grown under T3SS-inducing conditions in the presence of milk-derived peptone from a variety of brands (F- Formedium Limited, SC- Santa Cruz Biotechnology, M- Millipore Sigma, G- Gibco ThermoFisher Scientific), at aerobic conditions. The secreted fractions were normalized according to OD_600_, concentrated from the supernatants of bacterial cultures, and analyzed by western blotting with an anti-EspD antibody. Bacterial pellets were analyzed for Intimin and Tir expression. DnaK was used to verify equal bacterial loads.
